# Computer Vision-Based Bridge Inspection and Monitoring: A Review

**DOI:** 10.3390/s23187863

**Published:** 2023-09-13

**Authors:** Kui Luo, Xuan Kong, Jie Zhang, Jiexuan Hu, Jinzhao Li, Hao Tang

**Affiliations:** 1 College of Civil Engineering, Hunan University, Changsha 410082, China; luokui@hnu.edu.cn (K.L.); zhangjietm@hnu.edu.cn (J.Z.); jxhu123@hnu.edu.cn (J.H.); kingle@hnu.edu.cn (J.L.); tanghao200097@hnu.edu.cn (H.T.); 2 Key Laboratory for Damage Diagnosis of Engineering Structures of Hunan Province, College of Civil Engineering, Hunan University, Changsha 410082, China

**Keywords:** bridge inspection and monitoring, computer vision, surface defect detection, displacement measurement, modal identification, damage detection, vehicle parameter identification

## Abstract

Bridge inspection and monitoring are usually used to evaluate the status and integrity of bridge structures to ensure their safety and reliability. Computer vision (CV)-based methods have the advantages of being low cost, simple to operate, remote, and non-contact, and have been widely used in bridge inspection and monitoring in recent years. Therefore, this paper reviews three significant aspects of CV-based methods, including surface defect detection, vibration measurement, and vehicle parameter identification. Firstly, the general procedure for CV-based surface defect detection is introduced, and its application for the detection of cracks, concrete spalling, steel corrosion, and multi-defects is reviewed, followed by the robot platforms for surface defect detection. Secondly, the basic principle of CV-based vibration measurement is introduced, followed by the application of displacement measurement, modal identification, and damage identification. Finally, the CV-based vehicle parameter identification methods are introduced and their application for the identification of temporal and spatial parameters, weight parameters, and multi-parameters are summarized. This comprehensive literature review aims to provide guidance for selecting appropriate CV-based methods for bridge inspection and monitoring.

## 1. Introduction

Bridges play a key role in the transportation infrastructure system [1,2,3]. A bridge structure is subjected to the coupling effects of multiple factors such as wind, earthquakes, impact, and vehicle load. In addition, the degradation of the material itself will cause varying degrees of damage during the service period. Therefore, ensuring the safety and reliability of bridge structures is one of the important tasks in the operation, maintenance, and management of civil infrastructure systems [4,5,6].

Bridge inspection and monitoring are usually used to evaluate the status and integrity of bridge structures, which mainly include visual inspection, nondestructive detection, and health monitoring. The traditional visual inspection has difficulty meeting the inspection requirements of modern bridges, due to its relatively subjective decision-making process, low detection efficiency, and low safety. To improve the detection efficiency of bridge surface defects detection and promote the intelligence of the industry, many researchers have used cameras to collect images of bridge surface defects [7,8,9,10] and have combined deep learning (DL) and computer vision (CV) techniques to analyze the camera-recorded images intelligently [11,12,13,14,15,16]. In addition, vibration measurement is very important for evaluating the integrity and safety of bridges. The traditional vibration measurement method obtains the dynamic response of a bridge through sensors installed on the bridge. However, this method is a contact method, which has disadvantages such as difficulty in sensor installation, high equipment cost, and low testing efficiency. To overcome this problem, some scholars have proposed non-contact vibration measurement methods [17,18].

In recent years, with the continuous advancement of CV techniques and image acquisition equipment, CV-based bridge inspection and monitoring methods have emerged and have been validated in practical engineering applications [19,20,21,22,23]. They have attracted the attention of researchers and engineers due to their advantages, such as being low-cost, simple to operate, remote, and non-contact [24]. CV-based inspection and monitoring methods have been widely used in a variety of tasks for structural health monitoring (SHM), such as surface defect detection in bridges [25,26,27], vibration measurements [28,29,30], and vehicle parameter identification [31,32].

Some scholars have published related reviews of CV-based SHM. Zhou et al. [33] introduced popular DL-based crack segmentation algorithms and summarized some publicly available large-scale crack image datasets and popular performance metrics for crack detection. Deng et al. [34] reviewed the current state-of-the-art CV-based crack identification and quantification systems. Jeong et al. [35] summarized the CV-based crack detection algorithm and reviewed the application of unmanned aerial vehicles (UAVs) for crack detection. Zhuang et al. [36] reviewed the applications of CV techniques to structural deformation monitoring from two aspects, that of physical impact and target-tracking algorithm impact. Poorghasem et al. [37] summarized the robot-based approach to structural vibration measurement from two aspects of hardware and software, and discussed its challenges and opportunities. However, most of the above literature reviews focused on crack detection and deformation monitoring of civil engineering structures; there is a lack of systematic reviews on bridge inspection and monitoring based on CV techniques. To fill this gap, this study reviews state-of-the-art applications of CV techniques for bridge inspection and monitoring. The principles and general procedures of surface defect detection, vibration measurement, and vehicle parameter identification based on CV techniques are introduced, followed by a review of their applications. This review will help researchers to gain a rapid and systematic understanding of bridge inspection and monitoring based on CV techniques, and an idea of the challenges that still need to be overcome.

This review is organized as follows: Section 2 describes the principle of CV-based surface defect identification and summarizes its application to bridge inspection. Section 3 reviews the principle of CV-based vibration measurement and its application to the identification of displacement, modal parameters, and structural damage. Section 4 introduces the CV-based vehicle parameter identification methods and reviews their application to the identification of vehicles’ temporal, spatial, and weight parameters. Finally, Section 5 provides conclusions and future research prospects.

## 2. CV-Based Surface Defect Detection

Surface defect detection is an essential part of bridge inspection for the condition assessment, maintenance, and management of bridges. In recent years, CV-based methods for surface defect detection have developed rapidly and become a research hotspot [3,38,39,40].

### 2.1. General Procedure

**Figure 1:**
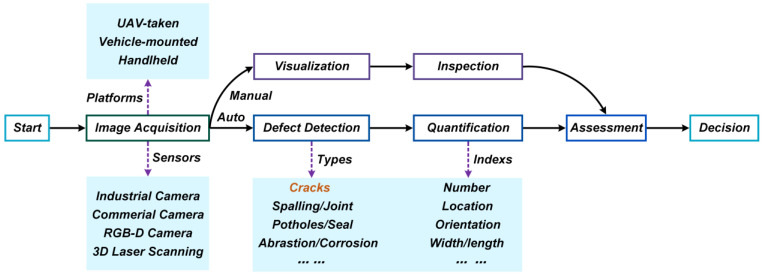
CV-based surface defect detection process for bridges [41].

Figure 1 shows the general procedure of CV-based surface defect detection, consisting of five steps, i.e., image acquisition, defect detection, quantification, assessment, and decision [41].

#### 2.1.1. Image Acquisition

The acquisition equipment for bridge surface defect images in the existing work is mainly divided into four categories: industrial cameras, commercial cameras, RGB-D cameras, and 3D laser scanners [41]. Industrial/commercial cameras are widely used for surface defect image acquisition on bridges due to their ease of deployment. However, industrial/commercial cameras cannot acquire a depth of information about the surface defects. To solve this problem, some scholars have proposed using an RGB-D camera to obtain a depth of information about surface defects. In addition, 3D laser scanning technology is also used to obtain surface defect images of bridges.

The collection platforms for bridge surface defects are divided into three categories: handheld, vehicle-mounted, and UAV-taken [41]. Smartphones are commonly used handheld devices. Compared with the handheld method, the vehicle-mounted cameras have the advantages of intelligence and high efficiency. However, the vehicle-mounted camera cannot be directly used to collect images of surface defects on piers, pylons, and cables. To solve this problem, some scholars have proposed a UAV-based surface defect image acquisition system.

#### 2.1.2. Defect Detection

The algorithms of object detection for CV-based surface defect detection are mainly categorized into two types: i.e., image processing algorithms and DL algorithms. The widely used image processing algorithms include the threshold algorithm, edge algorithm, and region algorithm.

- **Threshold algorithm**. The threshold method is the most commonly used image segmentation algorithm. Its principle is to divide the image into several different classes overall or locally based on one or more gray thresholds to separate the target and the background [42]. The threshold segmentation methods mainly include the gray histogram threshold method, the Otsu algorithm, and the iterative threshold segmentation method [43].
- **Edge algorithm**. The edge algorithm is a segmentation technology based on image discontinuity, which is often completed by convolution based on edge differential operators. According to different calculation methods of gradients, traditional edge detection operators can be divided into two categories: first-order derivatives (Sobel gradient operator, Canny gradient operator, Prewitt gradient operator) and second-order derivatives (Laplace operator). The Sobel operator and Canny operator are most widely used in the field of crack extraction [44,45].
- **Region algorithm**. Traditional edge detection methods are prone to breakage when extracting tiny cracks. Some scholars have studied the complete extraction methods of fracture connections. Therefore, a region-based crack extraction algorithm is proposed. The seed-growing algorithm is a common region-based algorithm [46].

The DL-based surface defect detection method does not require pre-definition of detect features or image preprocessing. Through a large number of samples for learning and automatic feature extraction, the identification and extraction of surface defects are realized on this basis. The DL algorithm can effectively eliminate the influence of structural surface noise and interference and extract surface defects more accurately. DL-based surface defect detection algorithms are divided into three categories: image classification algorithms, target detection algorithms, and image segmentation algorithms [47].

- **Image classification algorithm**. Convolutional neural networks (CNN) are the current mainstream image classification algorithm. This algorithm was first proposed by Lecun et al. [48] and applied to the recognition of handwritten digits. Its network structure consists of multiple convolutional layers and several fully connected layers. The input image enters the fully connected layer after convolution, pooling, activation, and other operations and finally outputs the classification result. Commonly used network structures include AlexNet [49], GoogLeNet [50], VGGNet [51] and ResNet [52].
- **Target detection algorithm**. The target detection algorithm needs to categorize and surround the object to be tested with a rectangular frame in the image. Target detection algorithms are divided into two categories: the first category is the candidate region-based algorithm represented by R-CNN [53,54]. The second category is the regression-based algorithm represented by YOLO [55,56]. The latter is called a single-stage object detector because it does not include a candidate region generation process and can directly obtain the classification and location of the object. Therefore, in terms of performance, single-stage object detectors are computationally faster and can achieve real-time detection, but their accuracy is worse than that of the two-stage object detector.
- **Image segmentation algorithm**. The fully CNN algorithm can achieve pixel-level segmentation of crack images. This method was first proposed by Long et al. [57]. Its network structure cancels the fully connected layer on the basis of the CNN so that it can address any size of input images. The image segmentation algorithm can directly complete the whole process of extracting cracks from the original image in the process of crack extraction [58,59]. The encoder–decoder structure represented by U-Net [60,61] is more widely used in this field, and the dilated/atrous convolution structure represented by Deeplab [62] is less used.

#### 2.1.3. Quantification

The quantification of surface defects is performed to determine the location, direction, length, and width of the defects. The methods for defect length measurement include the Euclidean distance method, chain code method, and skeleton line method [63]. The methods for defect width measurement include the average width method, centerline method, inscribed circle method, edge line minimum distance method, gray value method, and edge gradient method [64]. The methods for defect area measurement include the pixel equivalent method and approximate estimation method.

#### 2.1.4. Assessment and Decision

After obtaining the location and size of the surface defect of bridges through the identification and quantification algorithms, it is necessary to combine the historical data of the bridge condition and the bridge specification to evaluate the operation status of the bridge. According to inspection and monitoring data, it provides a scientific decision-making basis for bridge safety monitoring, operation maintenance and management [65].

### 2.2. Application of Surface Defect Detection in Bridges

Surface defect detection is of great significance to the safe operation and maintenance of bridges. For different types of defects, many researchers have developed corresponding detection methods using CV techniques.

#### 2.2.1. Concrete Cracks

**Figure 2:**
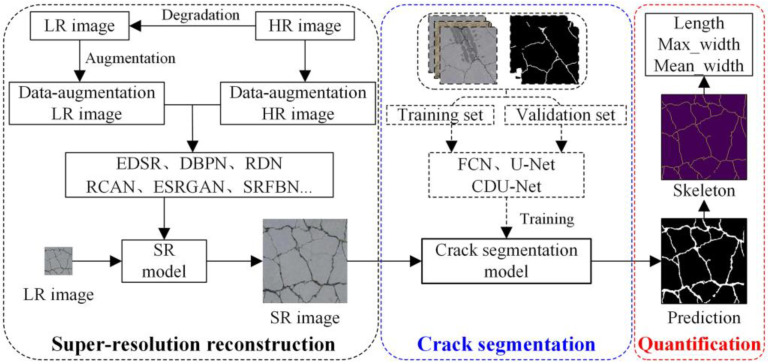
Framework for crack segmentation and feature quantification based on SR images [68].

Cracks are one of the most common surface defects of concrete bridges, reflecting the safety and durability of bridge structures. To ensure the safe operation of bridges, it is necessary to conduct regular detection of concrete cracks, which requires the development of accurate and rapid concrete crack detection methods. Li et al. [66] developed an automatic bridge crack detection system based on UAV and Faster R-CNN. Dung et al. [59] proposed a concrete crack detection method based on a deep fully convolutional neural (FCN) network. This method can only identify cracks, which are difficult to automatically quantify. Zhang et al. [67] proposed an automatic pixel-level crack detection method based on an improved U-net network (i.e., Crack U-net). However, Crack U-net cannot accurately detect cracks from low-resolution (LR) images. To solve this problem, Xiang et al. [68] proposed an automatic tiny-crack detection method based on super-resolution (SR) reconstruction and semantic segmentation, as shown in Figure 2. The SR image reconstructed using the DL-based model is input into the proposed semantic segmentation network for crack segmentation, and the crack length and width are measured according to the improved medial axis transformation method. Due to the lack of open-source crack datasets (the open-source bridge surface defect datasets are shown in Table 1), using the fully supervised segmentation method requires manual labeling of a large amount of data, which takes a long time. To solve this problem, Wang et al. [69] proposed a semi-supervised semantic segmentation network for crack detection. However, this method has poor ability to detect tiny cracks. To solve the problem of difficult segmentation of tiny cracks, Chu et al. [70] proposed a multi-scale feature fusion network with an attention mechanism to capture the local features of tiny cracks.

After identifying the cracks using crack detection algorithms, it is necessary to analyze and quantify the cracks, including their length and width. Dare et al. [75] used manual selection of the start and end points of the cracks to connect the cracks into a polyline, and calculated the crack width according to the threshold. Fujita et al. [76] applied median filtering and multi-scale linear filtering for noise reduction in the crack region, and calculated the crack width through a local adaptive threshold. Luo et al. [77] calculated the crack width using the minimum distance between two crack edges. Kim et al. [78] used the two closest edge points to the crack skeleton point to measure the crack width. Flah et al. [79] used the DL algorithm and the improved Otsu algorithm to quantify the crack length, width, and orientation angle. Liu et al. [80] combined VGG-16 and morphology to segment cracks at the pixel level and quantify their lengths and widths. Miao et al. [81] quantified the width and direction of cracks using the TaHE threshold algorithm after histogram equalization. Jahanshahi et al. [82] used the correlation values between a strip kernel and a subset of binary crack images to calculate crack width and orientation.

Most of the current research has focused on crack identification and segmentation algorithms. However, the development of cracks often reveals the damage mechanism of the structure, and the tracking and monitoring of existing cracks could be useful for analyzing the performance of bridge structures. Therefore, further research is required to determine how to accurately screen out and monitor cracks that affect structural safety.

#### 2.2.2. Concrete Spalling

The concrete spalling can result in exposed rebar, and the rebar is prone to rust when exposed to the air for a long time. Therefore, early detection of concrete spalling is necessary to ensure the integrity of the bridge structure. In recent years, many scholars have used the CV technique to detect concrete spalling. Cao et al. [83] proposed an automatic concrete spalling detection method based on LogitBoost classification and regression tree modeling. Santos et al. [84] proposed an automatic identification method for concrete spalling in reinforced concrete bridges based on AlexNet migration learning with an accuracy of 99.1%. Hoang et al. [85] used a Gabor filter to extract texture information of concrete spalling and a logistic regression model based on the state-of-the-art adaptive moment estimation to detect concrete spalling. However, the method was unable to detect minor spalling. To solve this problem, Hoang et al. [86] developed a method for detecting minor spalling of concrete surfaces based on image texture analysis and a novel jellyfish search optimizer. Nguyen et al. [87] enhanced the prediction performance of the extreme gradient boosting machine (XGBoost) using a meta-heuristic Aquila optimizer metaheuristic, and employed XGBoost and a deep convolutional neural network (DCNN) to categorize concrete spalling into shallow spall and deep spall. Abdelkader et al. [88] proposed an entropy-based method for detecting and evaluating the severity of concrete bridge spalling.

#### 2.2.3. Steel Structure Corrosion

In environments such as the ocean, hot and humid conditions, acid rain and salt spray, the surface of steel structures is extremely prone to corrosion. The main corrosion types of steel bridges include surface corrosion, cable, and suspender corrosion. In recent years, with the rapid development of CV technology and UAV technology, many scholars have applied the CV technique and DL algorithm to detect steel structure corrosion. Forkan et al. [89] proposed a framework for detecting corrosion of steel bridges based on UAV vision. Dong et al. [90] proposed a multi-vision scanning system for detecting corrosion on cable surfaces and used a panoramic image stitching processing algorithm to identify corrosion defects on the surface of the cables. Hou et al. [91] proposed an automatic detection method for surface defects of cables based on the Mask R- CNN network. Compared with other networks, the proposed method has higher applicability and accuracy. To improve the accuracy and speed of cable surface defect detection, Huang et al. [92] proposed an intelligent cable damage detection method based on CNN, which can realize the automatic extraction of cable surface image features. Khayatazad et al. [93] developed a corrosion detection algorithm for steel structures by combining two visual parameters, i.e., roughness and color, which can be used to effectively locate the corrosion areas. To realize the precise location of corrosion defects, Meng et al. [94] developed a lightweight DL model for cable corrosion detection by combining intelligent image recognition and magnetic memory technology. This method can realize the precise identification and localization of cable corrosion defects, and its detection accuracy is as high as 97.18%.

#### 2.2.4. Multi Defects

There are various types of bridge surface defects, and there may be overlap between different defects. However, traditional CNN can only detect a single defect. To solve this problem, Dunphy et al. [95] utilized transfer learning-based generative adversarial networks for multi-defect detection. Hüthwohl et al. [96] proposed a multi-classifier for reinforced concrete bridge defects. The classifier can reliably classify multiple defect types with an accuracy of 85%. Cha et al. [97] proposed a Faster R-CNN based multi-defect detection method for steel bridges, which can detect concrete cracks, steel corrosion with two levels (medium and high), bolt corrosion, and steel delamination. Compared with the traditional CNN network, Faster R-CNN has higher computational efficiency. To further improve the accuracy of multi-disease detection, Kim et al. [98] proposed a concrete damage detection method based on Mask R-CNN. The method can detect cracks, efflorescence, rebar exposure, and spalling with an accuracy of more than 90% in outdoor environmental tests. Zhang et al. [99] proposed a multi-defect detection method for bridges by combining YOLOv3 and transfer learning. The testing accuracy was greatly improved compared to the traditional YOLOv3. Li et al. [100] developed a multi-defect detection method for concrete bridges based on the FCN network. The method can detect cracks, spalling, efflorescence, and holes. It has good applicability in practical applications. However, the above method cannot realize the real-time detection of multiple defects. To solve this problem, Ali et al. [101] developed a real-time bridge multi-defect detection system based on UAV utilizing improved Faster R-CNN.

Researchers have proposed many CV-based methods to detect bridge surface defects. However, many problems in this field have not been adequately addressed. Some relevant challenges are summarized as follows:

(1) Due to the lack of open-source datasets of bridge surface defects, researchers need to perform time-consuming and labor-intensive work for data preparation. The establishment of public datasets with generalization capability is urgently needed to solve the automated bridge surface defect detection [41].
(2) CV-based surface defect detection system is susceptible to its own hardware and external environmental factors. That is, hardware and environmental factors can adversely affect the performance of the detection system [65].
(3) It is difficult for existing DL algorithms to realize real-time detection of bridge surface defects. The computational efficiency needs to be improved, although some improvement has been realized through lightweight DL models [102].
(4) Most of the current research focuses on how to utilize advanced algorithms to identify surface defects of the bridge, while the assessment of its severity requires further research.

### 2.3. Robotic Platforms

**Figure 3:**
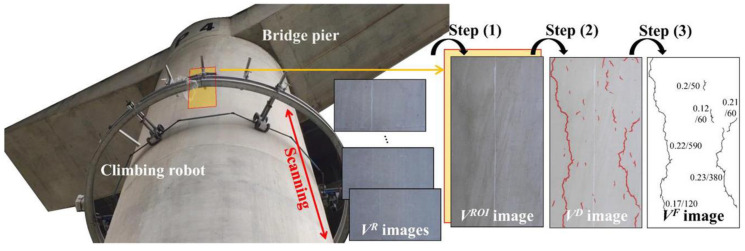
Inspection of bridge pier cracks using a ring-type climbing robot [109].

With the rapid development of artificial intelligence and robotics, various robotic platforms for bridge inspection have been developed. La et al. [103] integrated an advanced nondestructive testing technique into an automated robot to achieve accurate detection of bridge deck cracks. However, the robot was unable to detect cracks at the bottom of the bridge. Xie et al. [104] developed a new vehicle-based robotic inspection system with the ability to detect surface defects on the bridge deck and under the bridge. However, the ground-based inspection vehicle could not be directly used for defect detection of hard-to-reach structural components such as cables, bridge towers, piers, and bridge bearings. Therefore, further development of more complex robotic platforms with multiple locomotion capabilities is required. Leibbrandt et al. [105] designed a wall-climbing robot that can be attached to the bottom of a bridge to detect cracks. For the pier and abutment parts of a bridge, a wall-climbing robot can be utilized for crack detection [106,107,108]. In addition, Jang et al. [109] developed a ring-shaped wall-climbing robot (see Figure 3), which can realize the automatic detection of cracks on bridge piers at high altitudes, avoiding the safety hazards presented by working at high altitudes. Boomeri et al. [110] designed a cable-climbing robot with adaptive force control capability, which realizes the detection of the surface defect of cables. The existing bridge surface defects’ robot platforms are shown in Table 2.

Compared with traditional visual inspection methods, wall-climbing robots have the advantage of reducing worker safety risks and maintenance costs. However, the load-carrying capacity of the wall-climbing robot platform is relatively low, and few sensors can be integrated into the platform for defect detection. In addition, different wall-climbing robots need to be designed for different bridge components. Cable-climbing robots have been widely used to detect surface defects in cables. However, the cable-climbing robot can only detect one cable at a time, which limits the detection efficiency. Compared with ground-mobile robots, wall-climbing and cable-climbing robots, UAVs have the unique advantage of approaching some difficult-to-reach regions of bridges. One of the bottlenecks with regard to applying UAVs to engineering practice is the limited power provided by lithium-ion batteries for a short duration. Another problem is how to locate the UAV in environments where GPS is unavailable.

Different robot platforms for surface defect detection have been developed for different bridge components. The development of a robot platform for surface defect detection with many applicable scenarios and low cost will be the focus of future research.

## 3. CV-Based Vibration Measurement

Vibration measurement is a key part of bridge SHM. The vibration displacement and modal parameters of the bridge can be obtained through vibration measurement. The modal parameters are important indicators to reflect the health of bridges, and damage identification and performance evaluation of bridges can be performed through the changes in modal parameters. The traditional vibration measurement method depends on contact sensors. This method has the disadvantages of high cost, difficult sensor installation, low precision, and poor real-time performance, which makes it difficult to meet the demand for real-time monitoring of bridge dynamic response. In recent years, CV-based vibration measurement methods have developed rapidly. Due to the advantages of non-contact, full-field measurement, and strong real-time performance, it has been widely used in displacement measurement and modal identification.

This section introduces the general procedure of CV-based vibration measurement, followed by a review of applications in displacement measurement, modal identification, and damage identification.

### 3.1. General Procedure

**Figure 4:**
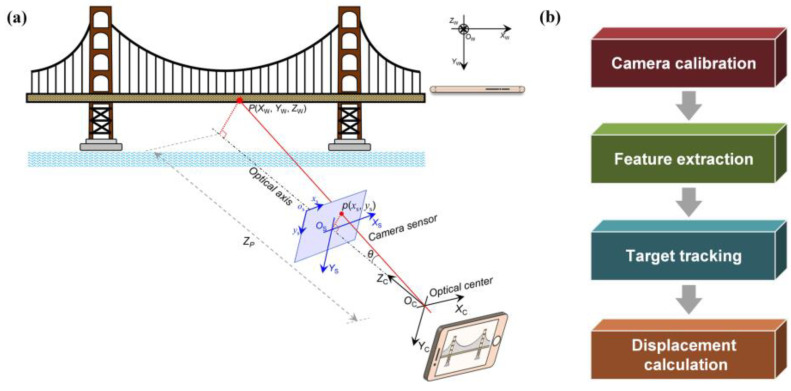
The flow chart of CV-based bridge displacement identification [124]: (**a**) CV-based displacement measurement principle; (**b**) basic steps for determining displacement.

The CV-based vibration measurement mainly includes four steps: camera calibration, feature extraction, target tracking, and displacement calculation [65,123], as indicated in Figure 4.

#### 3.1.1. Camera Calibration

The main purpose of camera calibration is to explore the projection relationship from 3D world coordinates to 2D image coordinates, thereby realizing the transformation of each point in the image to the 3D world. In the process of projection, the image will have central perspective distortion [125] and radial distortion [126], and camera calibration is also implemented to correct the image distortion.

- **General camera calibration**. Camera calibration needs to estimate the intrinsic parameters, distortion coefficients, and extrinsic parameters of the camera. The intrinsic parameters and distortion coefficients are determined by the lens of the camera, and the extrinsic parameters are determined by the position and direction of the camera. During the camera calibration process, the external matrix and internal matrix need to be estimated.

The coordinates in the 3D world are projected to the 2D image coordinates through the camera, and the transformation expression is as follows:

(1) $$s\left(\begin{array}{c} x\\ y\\ 1 \end{array}\right)=\left[\begin{array}{ccc} f_{x} & \gamma & c_{x}\\ 0 & f_{y} & c_{y}\\ 0 & 0 & 1 \end{array}\right]\;\left[\begin{array}{llll} r_{11} & r_{12} & r_{13} & t_{1}\\ r_{21} & r_{22} & r_{23} & t_{2}\\ r_{31} & r_{32} & r_{33} & t_{3} \end{array}\right]\left(\begin{array}{c} X\\ Y\\ Z\\ 1 \end{array}\right)$$

The simplified expression of Equation (1) is as follows:

(2) $$sx=K\left[R|t\right]X$$

where *s* is the scale factor and $x={\left(x,y,1\right)}^{T}$ are 2D image coordinates, $X={\left(X,Y,Z,1\right)}^{T}$ are 3D world coordinates, ***K*** is the camera intrinsic parameter, and ***R*** and ***t*** are camera extrinsic parameters. In the intrinsic matrix, $f_{x}$ and $f_{y}$ are the focal length of the lens in the horizontal and vertical directions, $c_{x}$ and $c_{y}$ are the offset of the main axis in the horizontal and vertical directions, and γ is the skew factor of the lens.

It can be seen in Equations (1) and (2) that the intrinsic parameters of the camera are related to the camera and the lens, while the extrinsic parameters of the camera are related to the relative position of the camera and the objects. The intrinsic parameters will not change unless the lens focal length and other hardware parameters change. However, the extrinsic parameters of the camera should be calibrated for different application scenarios. The black-and-white chessboard is often used for camera calibration. Commercial software such as MATLAB 2019a and Ni Vision 2020 and the open-source library OpenCV 4.2.0 provide a wealth of toolkits that can be used to achieve rapid calibration.

- **Homography matrix method**. When using a camera for displacement measurement in a 2D structure plane, engineers simplify the camera calibration process in terms of the camera, lens, and motion characteristics of the tested structure. That is, the homography matrix method is used for camera calibration [127], and Equation (1) can be simplified as

(3) $$s\left(\begin{array}{c} x\\ y\\ 1 \end{array}\right)=\left[\begin{array}{ccc} h_{11} & h_{12} & h_{13}\\ h_{21} & h_{22} & h_{23}\\ h_{31} & h_{32} & h_{33} \end{array}\right]\;\left[\begin{array}{c} X\\ Y\\ 1 \end{array}\right]$$

(4) $$H=\left[\begin{array}{ccc} h_{11} & h_{12} & h_{13}\\ h_{21} & h_{22} & h_{23}\\ h_{31} & h_{32} & h_{33} \end{array}\right]$$

where ***H*** is the homography matrix.

**Figure 5:**
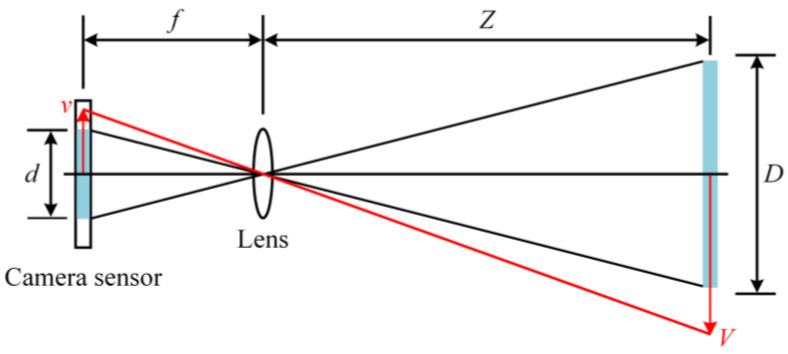
Scaling factor calculation diagram.

- **Scaling factor method**. The above method can be further simplified by using the pinhole camera model to estimate the scale factor if the lens distortion is small enough to be ignored. An illustration of scale factor estimation is shown in Figure 5.

When the optical axis of the camera is perpendicular to the structure plane, that is, the optical axis is collinear with the normal of the structure plane, the scale factor *S* is determined by

(5) $$S=\frac{D}{d}=\frac{Z}{f}\cdot d_{pixel}$$

where *D* is the physical length of the selected object in the structure plane, *d* is the length in pixels of its corresponding image part, *f* is the focal length, *Z* is the distance from the camera lens to the structure plane, *V* is the physical displacement, *v* is the displacement in pixels of the image, and *d_pixel_* is the pixel size.

**Figure 6:**
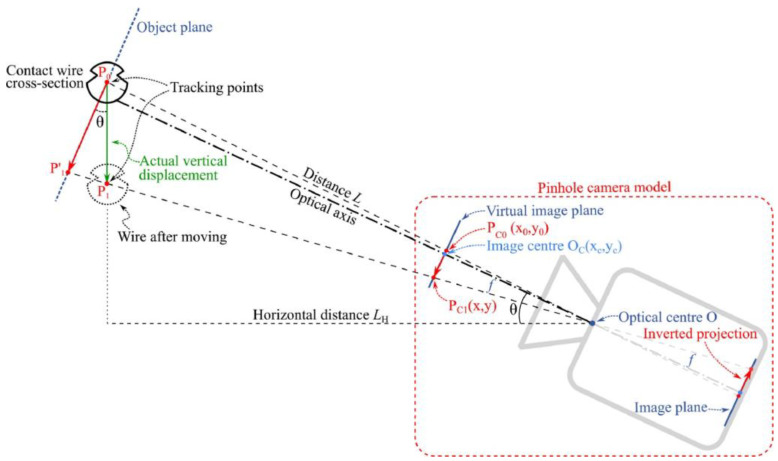
A pinhole camera model to estimate the scale factor considering the optical axis not perpendicular to the structure plane [29].

When the optical axis of the camera is not perpendicular to the structure plane, i.e., there is an angle θ between the optical axis and the normal to the structure plane, as shown in Figure 6. Jiang et al. [29] proposed a pinhole camera model to estimate structural displacement, compensating for the effect of angle θ. The scale factor $S^{\prime }$ in Equation (5) can be determined by

(6) $$S^{\prime }=\frac{Z}{f\cos^{2}\theta }\cdot d_{pixel}$$

#### 3.1.2. Feature Extraction

Image features are the basis for target tracking. When using CV technology to measure structural vibration, it is necessary to select and extract image features to facilitate the selection of subsequent tracking algorithms. Common image features include grayscale features [128], feature points [129], gradient features [130,131], shape features [132], color features [133], color or grayscale histograms, and image convolution blocks [134].

#### 3.1.3. Target Tracking

The task of target tracking is to track the tested structure or its markers according to the selected image features, so as to determine the position of features in each frame of the video or image sequence, and then obtain the vibration time history of the structure. Typical tracking methods used in displacement measurement include template matching [135], feature point matching [136], full-field optical flow [137], sparse optical flow [138], geometric matching [132], particle image velocimetry [139], color matching [133], and DL-based target-tracking algorithms [140].

A comparison of different target-tracking algorithms is shown in Table 3. The field conditions listed in Table 3 are the weather conditions of the experiment. The measurement error of the template matching algorithm is the largest, followed by the optical flow method, while the error of the feature matching method is the smallest.

#### 3.1.4. Displacement Calculation

The pixel displacement of the measured structure in the image is obtained via image tracking. After that, the intrinsic and extrinsic parameter matrix in Equation (1), the homography matrix in Equation (4), or the scale factor in Equation (5) are used to convert the pixel displacement in the image into the physical displacement in the real world.

### 3.2. Displacement Measurement

The CV-based displacement measurement method has advantages such as remote and non-contact, and thus has been rapidly developed in the field of bridge engineering in recent years. Many CV-based displacement measurement systems have been developed for different application scenarios [128,151,152,153,154,155,156,157].

#### 3.2.1. Displacement Measurement Based on a Fixed Camera

**Figure 7:**
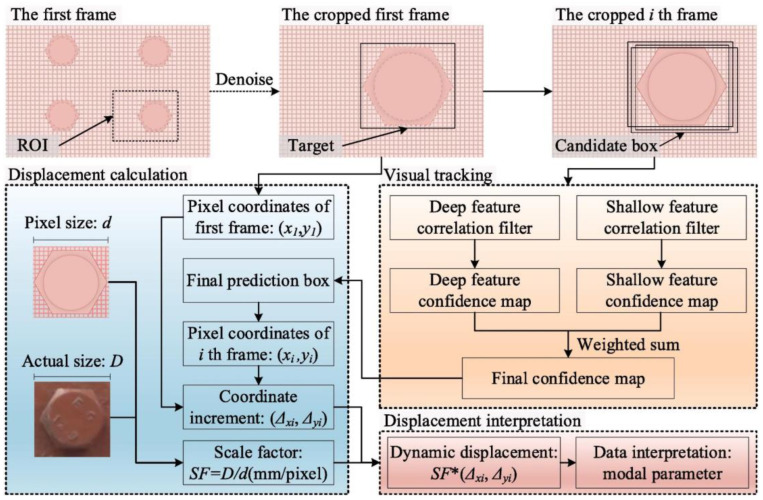
Framework for target-free displacement measurement [162].

In the 1990s, Stephen et al. [158] began to apply non-contact vision sensing technology to the vibration displacement measurement of the Humber Bridge in the UK. Subsequently, many scholars applied template-matching algorithms, optical flow methods, digital image correlation (DIC), and DL-based target-tracking algorithms to bridge vibration displacement measurement. Guo et al. [159] proposed a dynamic displacement measurement method based on the Lucas–Kanade template tracking algorithm, and the effectiveness of the method in dynamic displacement measurement was verified by the measurement results of a viaduct. Ye et al. [160] proposed a multi-point displacement measurement system based on a color pattern-matching algorithm, and verified the accuracy and reliability of the system through field testing of arch bridges. To overcome the influence of environmental and operating conditions, Wang et al. [161] proposed novel gradient-based matching via a voting technique, which can provide reliable tracking of moved targets with different degrees of feature loss. To realize the target-free measurement of vibration displacement, Dong et al. [130] proposed a displacement measurement method based on the optical flow method. Zhu et al. [162] developed a structural dynamic displacement measurement method integrating a multi-resolution depth feature framework, as shown in Figure 7. This method uses the structural natural texture as the tracking region, which effectively avoids the installation problem of artificial targets. Dong et al. [123] converted the displacement obtained by feature matching into acceleration, and used the acceleration to evaluate the vibration serviceability of footbridges. Subsequently, Dong et al. [163] successfully identified the bridge’s displacement influence line using a template-matching algorithm based on the normalized cross-correlation coefficient. Javh et al. [164] applied the gradient-based optical flow method to identify the full-field displacement of steel beams, and the displacement identification accuracy was less than 0.001 pixels. Subsequently, in order to reduce costs, Javh et al. [165] developed a measurement system that can measure high-frequency vibration using a low-speed camera. Xie et al. [166] proposed a new edge detection operator to measure the vibration displacement of the cables under different illuminations. Compared with the Canny operator, this operator has stronger robustness under different illumination. Miao et al. [167] proposed a powerful and accurate phase-based 2D motion estimation method with strong anti-noise performance.

In addition, some CV-based methods for 3D displacement measurement of bridges have been developed. Helfrick et al. [168] applied 3D DIC to the measurement of structural vibration displacement for the first time. The results show that 3D DIC has great potential in the full-field vibration measurement of structures. Warren et al. [169] used a 3D point tracking algorithm to determine the 3D displacement of the structure. Pan et al. [170] reviewed the application of stereo-DIC in the full-field 3D displacement measurement of structures. Barone et al. [171] proposed a low-speed single-camera stereo-DIC system to obtain the 3D full-field vibration displacement of a cantilever beam. Shao et al. [172] developed a target-free full-field 3D displacement measurement based on a binocular vision system.

Existing target-tracking algorithms are only suitable for scenarios with a large amplitude of vibration displacement, and struggle to obtain small vibration measurements of bridge structures. However, the amplitude of vibration displacement of rigid structures such as small and medium-span bridges, short cables, and short suspenders under environmental excitation is very small, and it is difficult for general target-tracking algorithms to accurately obtain the displacement time history of bridge structures based on small vibrations.

#### 3.2.2. UAV-Based Displacement Measurement

In recent years, UAVs were introduced into the displacement measurement of bridges as mobile platforms with cameras installed. UAV technology has become an effective alternative to solve long-distance measurements. However, the vibration of the UAV itself will be coupled to the motion of the target, which reduces the measurement accuracy of the displacement. To solve this problem, many studies have been conducted to eliminate the UAV vibration by fixing targets, adding sensors to the UAV, or using a high-pass filter signal processing method. Han et al. [173] developed a vibration displacement measurement system based on UAV vision and used laser light to eliminate the vibration of the UAV. Tian et al. [174] proposed a cable vibration displacement identification method based on the UAV and line segments detector, and used the displacement subtraction of two points to eliminate the UAV vibration. To further improve the identification accuracy of the cable displacement, Zhang et al. [175] used the empirical mode decomposition algorithm to eliminate the UAV vibration. Liu et al. [176] developed a bridge vibration displacement measurement system based on UAV and DIC, and corrected the deviation caused by UAV vibration through a homography matrix. Jiang et al. [177] proposed a bridge vibration displacement measurement method based on a DL-based target tracking method and a dual-camera UAV system. When using their method, deformation point and stable point on the bridge are simultaneously captured by dual cameras with telephoto and wide-angle lenses, and the vibration displacement of the bridge and UAV is measured simultaneously, and the displacement of the UAV is eliminated by using the homography between the two cameras.

#### 3.2.3. Displacement Measurement Based on Visual Displacement and Acceleration

As displacement measurement based on the CV technique is usually limited to a low sampling rate, some scholars have proposed fusing vision-based displacement and acceleration to estimate displacement. Smyth et al. [178] proposed a multi-rate Kalman filtering method to identify displacements by the aggregating of displacements sampled at low frequency and accelerations sampled at high frequency. However, this Kalman filtering method is only applicable when the vision and acceleration measurements are synchronized, and the ratio of the sampling rates is an integer. To overcome this problem, Ma et al. [179,180] improved the multi-rate Kalman filtering method and proposed an adaptive multi-rate Kalman filtering method for the fusion of vision displacement and acceleration. Park et al. [181] applied complementary filtering to fuse vision displacement and acceleration to estimate displacement. Wu et al. [182] verified the feasibility of using complementary filtering in the fusion vision displacement and acceleration in field applications. However, the above methods are only suitable for obtaining high-fidelity displacements at sparse locations and cannot be used to obtain the full-field displacement of the bridge. To address this problem, Wu et al. [183] proposed a sparse accelerometer-aided CV technique and used the modal superposition method to obtain the full-field dynamic displacement through acceleration at sparse locations.

The existing bridge displacement monitoring methods based on CV techniques have problems such as a limited camera sampling rate and insufficient resolution in engineering applications. The development of a bridge displacement monitoring method based on the fusion of vision displacement and acceleration requires further research.

### 3.3. Modal Identification

Modal parameters (i.e., natural frequency, mode shape and damping ratio) are important indicators to reflect the structure status. The changes in the modal parameters can be used to identify bridge structural damage and evaluate the performance of the bridge structure [143,184,185,186,187]. In this section, a CV-based method for identifying bridge modal parameters is reviewed.

#### 3.3.1. Frequency Identification

**Figure 8:**
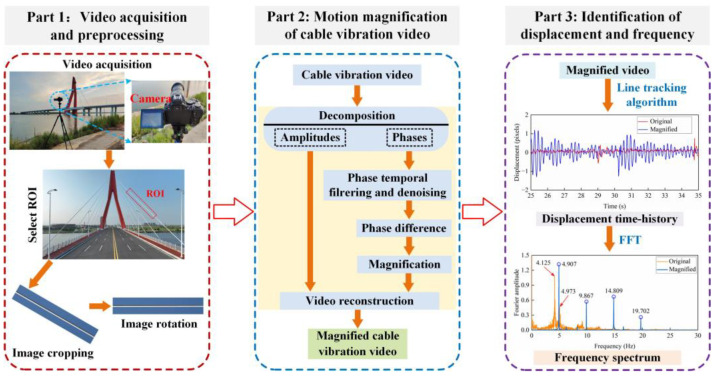
A framework for cable vibration measurements based on CV techniques [191].

Yoon et al. [188] investigated the potential of consumer-grade cameras for structural modal identification using a CV-based target-free method and an eigensystem realization algorithm. Feng et al. [151] proposed a bridge frequency identification method based on an upsampling cross-correlation template matching algorithm, and verified the feasibility of using vision technology to identify bridge frequencies through field tests. Ji et al. [189] used the Canny edge detection operator for image processing, obtained the displacement response from the image sequence reconstruction, and then obtained the vibration frequency of the structure according to the wavelet transform. Dong et al. [128] proposed a multi-point synchronous method for measuring structural dynamic displacement, and identified the natural frequencies of simply supported rectangular steel beams via FFT. Xu et al. [141] used the zero-mean cross-correlation template-matching algorithm to monitor the dynamic displacement of a cable-stayed bridge under the action of crowds, and analyzed the changes in the instantaneous frequency and vibration amplitude of the bridge when large crowds crossed the bridge. Ngeljaratan et al. [190] used DIC to monitor the vibration displacement of a steel truss pedestrian bridge and obtained the vibration frequency of the pedestrian bridge through signal processing technology. Feng et al. [21] proposed a non-contact cable natural frequency test method based on the orientation code matching algorithm. Hoskere et al. [148] proposed using UAV to obtain the natural frequencies and mode shapes of full-scale infrastructure, which solved the problem of difficult modal identification of full-scale infrastructure. However, the above methods make it difficult to measure the small vibrations of the bridge structures. To solve this problem, Luo et al. [191] proposed a method for measuring small vibrations of cables based on a broad-band phase-based motion magnification and line tracking algorithm, as shown in Figure 8. The results show that using the broad-band phase-based motion magnification algorithm to magnify small vibrations can significantly improve the identification accuracy of the cable frequency.

#### 3.3.2. Mode Shape Identification

**Figure 9:**
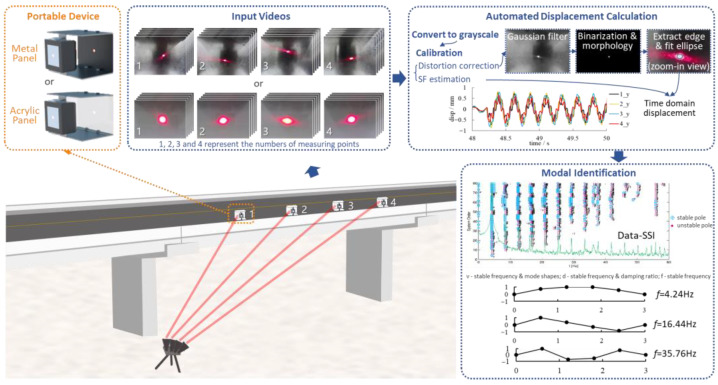
Bridge mode shape identification based on laser-and-camera system [198].

In recent years, the rapidly developed phase-based motion magnification (PMM) algorithm has been used for bridge mode shape identification [192]. Chen et al. [193] used the PMM algorithm and edge detection algorithm to obtain the mode shapes of cantilever beams, and performed an application study on a steel truss bridge [149]. Yang et al. [194,195] used the PMM algorithm as a post-processing step to visualize the mode shapes by magnifying the motion in a narrow frequency band around the natural frequencies of each order. Molina-Viedma et al. [196,197] combined the PMM algorithm and DIC to identify the mode shape of the cantilever beam. The results show that the combination of the PMM algorithm and DIC is suitable for the identification of structural modal parameters under high-frequency vibration, which makes up for the low precision of DIC when identifying structural high-frequency vibration displacement. To solve the difficulties posed by modal identification of full-scale bridge structures, Hoskere et al. [148] proposed a UAV-based vision-based mode shape identification method for full-scale suspension bridges. Han et al. [198] proposed a portable laser-and-camera system (as shown in Figure 9) for bridge displacement measurement, and used the Data-SSI method to identify bridge mode shapes. The system is easily assembled in the field and avoids the problems associated with the use of telephoto lenses.

In addition, full-field mode shape identification has also been investigated. Willems et al. [199] exploited the high spatial density of CV-based measurements to identify full-field mode shapes. Javh et al. [200] used the Least-Squares Complex-Frequency method combined with the Least-squares Frequency-domain method to identify the high-frequency full-field mode shape of steel beams. Zaletelj et al. [201] used frequency-domain triangulation to identify full-field modal shapes. Gorjup et al. [202] developed a new single-camera multi-view operating deflection shape (ODS) measurement system. This system uses only a single monochrome high-speed camera to achieve full-field 3D ODS measurements. Bhowmick et al. [203] used the optical flow method to track the pixel-level edge points of the structure, and obtained the full-field mode shape of the cantilever beam through the dynamic mode decomposition method. However, this method cannot obtain sub-pixel edges of structures. To solve this problem, Kong et al. [204] proposed a full-field mode shape identification method based on sub-pixel edge detection and edge tracking.

Most of the methods in the existing literature can only obtain the mode shape profile of the bridge and cannot perform quantitative analysis of the mode shape. In addition, the existing research mainly focuses on the identification of discrete mode shapes of bridges, and the identification of full-field mode shapes is in need of further research.

#### 3.3.3. Damping Identification

In the dynamic design of bridges, the damping ratio of the bridge needs to be estimated to evaluate the performance of the bridge under dynamic loads (i.e., seismic and wind loads). Monitoring of the bridge damping ratio is also crucial during bridge operation. Siringoringo et al. [18] used the PMM algorithm, discretized centroid searching algorithm, and dynamic mode decomposition to identify the damping of cantilever beams. Subsequently, Wangchuk et al. [205] applied this method to identify the damping ratio of the cable.

### 3.4. Damage Identification

The change in the boundary conditions and stiffness of the bridge structure will cause a change in the modal parameters, and the damage to the bridge can be identified through the change in modal parameters.

**Figure 10:**
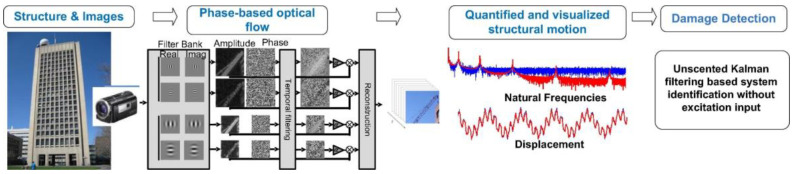
Principles of video-based damage detection [207].

With regard to bridge damage detection based on CV technology, Feng et al. [22] measured the displacement of multiple points of a test girder, and identified the damage to the test girder through the changes in the mode shape observed before and after the damage. Xu et al. [206] projected a visible laser line onto the damaged girder, extracted the curvature mode by tracking the laser line in the modal test, and determined the location and size of the damage according to the change in the curvature mode. Cha et al. [207] used the unscented Kalman filter to denoise the displacement obtained via the phase-based optical flow, and detected the boundary damage of the cantilever girder by identifying the stiffness and damping coefficient, as shown in Figure 10. Khuc et al. [208] determined the relationship between load (input) and response (output) based on CV technology and used the unit influence surface (UIS) to identify the damage to the bridge structure. Zhang et al. [209] proposed a novel vibration-based method to detect structural damage by combining phase-based motion estimation and CNNs. Shu et al. [210] proposed a damage identification method based on the DL algorithm. The method combines a data-driven approach with finite element model updating to quantify the damage location and damage level of structures. Hu et al. [211] proposed a hybrid method for damage detection and condition assessment of hinged joints of hollow slab bridges based on physical models and vision measurements.

Most of the research on bridge damage detection based on the CV technique was limited to the laboratory model test. The use of CV technique for real bridge damage detection is in need of further research.

## 4. CV-Based Vehicle Parameter Identification

Vehicle parameters are important evidence that reflects the stress state and traffic density of the bridge. The acquisition of the temporal and spatial distribution of vehicles on the bridge and the real-time monitoring of overloaded vehicles are essential in the field of bridge maintenance, management, and reinforcement.

This section introduces CV-based vehicle detection and tracking methods, followed by a review of applications in temporal and spatial, weight parameter and multi-parameter identification.

### 4.1. Vehicle Detection and Tracking Methods

**Figure 11:**
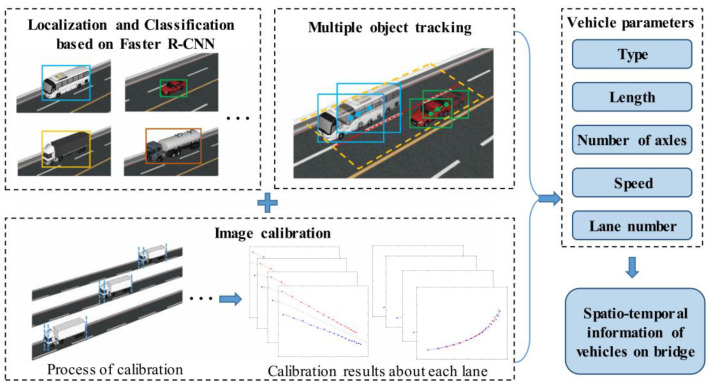
Framework for vehicle parameter identification [212].

The detection and tracking of moving vehicles is used to obtain the vehicle information through feature extraction and identification of the vehicle target in the moving state, and this process is shown in Figure 11. The moving vehicle detection methods based on CV and DL can be divided into three stages: moving object detection methods, object instance detection/segmentation methods, and fine-grained detection methods.

#### 4.1.1. Moving Object Detection Methods

Moving object detection has a wide range of applications in the field of intelligent transportation, which is the premise of obtaining vehicle information. By judging whether there is a moving vehicle in the image, the target is identified and its location is revealed. Information such as vehicle speed and vehicle distance can also be obtained through tracking analysis. Vehicle-moving object detection aims to extract moving vehicle targets in a video sequence for the subsequent tracking, identification, and analysis of moving vehicles. Moving object detection methods mainly include the inter-frame difference method [213,214,215,216,217], background subtraction method [218,219], and optical flow method [220].

#### 4.1.2. Object Instance Detection/Segmentation Methods

After the moving object detection method described above has been used to realize the identification and tracking of the target object, it is necessary to use the target instance detection method to conduct an in-depth analysis of the video-framed image. Further, it can be used to extract the multi-level feature information of the target vehicle, such as vehicle type, the spatial position of the vehicle on the bridge, vehicle spacing, license plate, and other feature parameters. Compared to the traditional methods, the DL-based target instance detection method can highly improve the accuracy and efficiency of detection, which has become the current mainstream method. Target instance detection methods are mainly divided into single-stage methods [221,222], two-stage methods [223,224], and multi-method fusion [225,226].

#### 4.1.3. Fine-Grained Detection Methods

Although DL-based target detection can extract multi-level feature information of moving targets, the difference in the appearance of the same type of vehicle is very small in the video image, and its subtle differences make it difficult to identify and track accurately. Fine-grained image identification, also known as the sub-category image classification method, has been highlighted as a promising technique in recent years. A more detailed sub-category division is useful for the identification of subtle differences that cannot be identified using the normal detection method. According to the manual intervention image information ratio, it can be divided into a strongly supervised fine-grained image identification model [227,228] and a weakly supervised fine-grained image identification model [229,230,231].

Strongly supervised fine-grained image identification has high classification accuracy, but requires a large amount of manual annotation information, and has poor practicability. Weakly supervised fine-grained image identification is an end-to-end identification mode, which has high identification accuracy and does not require human intervention, but the identification efficiency is relatively low. Commonly used strongly supervised fine-grained models include the Part-based R-CNN model and Pose Normalized CNN model. Weakly supervised fine-grained image identification models include Two-Level Attention models, Constellations models, and Bilinear CNN models.

### 4.2. Temporal and Spatial Parameters

#### 4.2.1. Temporal Parameter Identification

The time-related parameter of the vehicle mainly refers to the vehicle’s moving speed. The identification of vehicle speed can be achieved using the object detection method.

With regard to vehicle speed detection, some scholars have used background subtraction and optical flow methods to detect vehicle-moving targets. Jeyabharathi et al. [232] proposed a dynamic background subtraction based on the Diagonal Hexadecimal Pattern, which used an improved dynamic background subtraction and target-tracking algorithm to detect vehicle speed. The results showed that the method is simple to operate, insensitive to light, and has strong real-time performance. However, its detection accuracy is low. Doğan et al. [233] found enough vehicle feature reference points in each frame of the image and used sparse optical flow technology to estimate the real-time speed of a single vehicle or multiple vehicles. Hua et al. [234] presented a detect-then-track paradigm vehicle tracking model, in which the data-driven and optical flow tracking algorithms were employed to achieve vehicle speed estimation. The optical flow method does not require a priori information to recognize high accuracy and can recognize the motion information and background dynamic information of the target vehicle. However, the algorithm has poor anti-noise performance, and the calculations are complex, making this algorithm incapable of tracking moving targets in real-time.

**Figure 12:**
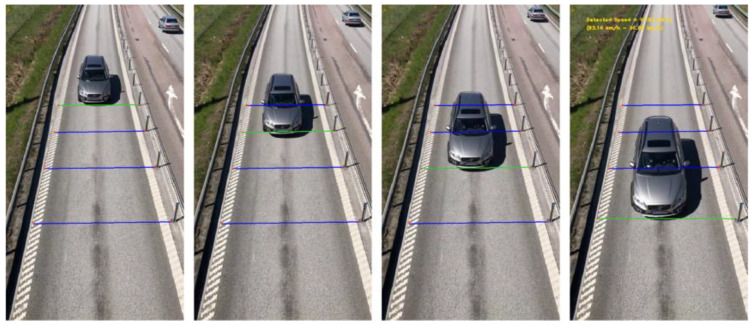
Vehicle speed detection [236].

Some scholars combined multiple methods to realize real-time vehicle speed recognition. Lan et al. [235] employed an improved three-frame difference method to extract the contour of the moving vehicle and used the gray constraint optical flow algorithm to obtain the optical flow value of the vehicle contour, and then identified the vehicle speed based on the ratio of the moving vehicle pixel to the road width. Javadi et al. [236,237] applied the frame rate of the camera, the positions of the four intrusion lines, and the motion pattern vector to establish the probability density function of the vehicle speed estimation, as shown in Figure 12. Compared with a vehicle equipped with GPS, the average error is within 1.77%. This shows that the vehicle speed measurement combined with multiple methods has high accuracy and robustness.

#### 4.2.2. Spatial Parameter Identification

Vehicle spatial parameters include the lane in which the vehicle is located, the longitudinal and lateral positions of the vehicle on the bridge, and the distance between vehicles.

- **Lane detection**. With the rapid development of computer technology, the DL algorithm is widely used in lane detection, and its detection accuracy has been greatly improved [238]. Li et al. [239] combined a multitask deep convolutional network with a recurrent neural network (RNN) to simultaneously detect the position and attribute of the lane in the image. Kim et al. [240] added a CNN to the random sample consensus (RANSAC) algorithm to eliminate the influence of edge noise, such as roadside trees and fences, in the road scene and enhanced the robustness of lane detection. Lee et al. [241] combined the features of road vanishing points with DL to form an end-to-end vanishing point guided network (VPGNet) for lane and road marking detection. The network was guided by vanishing points, which can effectively solve the problem of lane and road marking detection on rainy days and low illumination conditions.
- **Vehicle location identification**. For the identification of vehicle positions on the bridge, Chen et al. [242] employed template matching and PFA to track the moving vehicle on the bridge to identify the temporal and spatial distribution of bridge traffic in real time. The results showed that the feature-based and area-based hybrid approach of template matching could improve matching accuracy for lane-changing vehicles. Brown et al. [243] proposed a system that can identify a vehicle on a bridge and track its location through multiple video frames. The vehicles can be tracked along a bridge with acceptable errors in the location output. Ojio et al. [244] determined the position and axle spacing of the vehicle from the camera surveillance video and used the Lukas–Kanade algorithm to track the motion state.
- **Vehicle distance measurement**. Regarding the distance detection of vehicles, Kim et al. [245] proposed a new stereo-vision vehicle distance estimation method by combining the two-vehicle distance estimation methods of vehicle position and vehicle width. Park et al. [246] proposed the use of the size and position of vehicles in the image to estimate the virtual lanes, which can be used for inter-vehicle distance estimation when lane markings are not visible. To guarantee the safety of vehicles and reduce collision accidents, Chen et al. [247] proposed a vehicle front distance detection algorithm based on single-camera and dual-camera switching. By switching between a single camera and dual camera, the shadow image of the vehicle bottom, the characteristics of the vertical direction of the vehicle, the vehicle taillights and other characteristics are obtained to lock the position in front of the vehicle.

### 4.3. Weight Parameters

The rapid and accurate identification of vehicle weight is of great importance for the management and control of vehicle overload and the evaluation of road/bridge usage. The Bridge Weigh-in-Motion (BWIM) technique combined with cameras has been widely used in traffic load monitoring. BWIM is used to obtain vehicle load information, and cameras are used to determine the temporal and spatial distribution of vehicle loads on the bridge. In recent years, some scholars have also applied CV techniques to the identification of vehicle weight parameters [243,248].

#### 4.3.1. Weight Identification Based on Bridge Response

The non-contact BWIM method [249,250] uses bridges to measure vehicle weights without installing any sensors on the bridge. It has the advantages of convenient installation without interrupting traffic, causes no damage to the road, and allows real-time fast weighing. Ojio et al. [244] proposed a CV-based non-contact BWIM method, which requires two cameras to work together. One camera is used to measure sub-millimeter bridge deflections, and the other is used to monitor traffic and determine axle spacing. Ding et al. [251] proposed a vehicle load and load centroid measurement system based on the CV and vertical displacement of the body. According to the vertical characteristic distance recognized by the side camera, the vehicle load value can be obtained by resolving the parameters. Chen et al. [242] presented a method to identify the temporal and spatial distribution of vehicle loads for long-span bridges. The vehicle weight information is obtained through the BWIM system, and then the vehicle loads are tracked using CV techniques. The effectiveness and accuracy of the algorithm were verified by the field test on Hangzhou Bay Bridge. Micu et al. [252] employed adaptive thresholding and morphological reconstruction methods to extract vehicle length information from traffic videos. Further, they used statistical methods to establish the correlation between vehicle length and the weight measured by the BWIM system. Zhou et al. [253] divided the vehicle database into nine types of vehicle axle weight distribution intervals, and established the relationship between vehicle types and corresponding weight information. The vehicle type was accurately identified by using a deep convolutional neural network (DCNN), such as to obtain the corresponding vehicle weight. Dan et al. [254] proposed an information-fusion-based method for load identification to be applied to bridges of different lengths. In this method, the pavement-based weigh-in-motion system (WIMs) was installed at the entrance of the bridge to determine the weight of vehicles. The videos of traffic flow acquired by multiple cameras arranged along the bridge were employed to calculate each vehicle’s trajectory and location.

#### 4.3.2. Weight Identification Based on Tire Deformation

**Figure 13:**
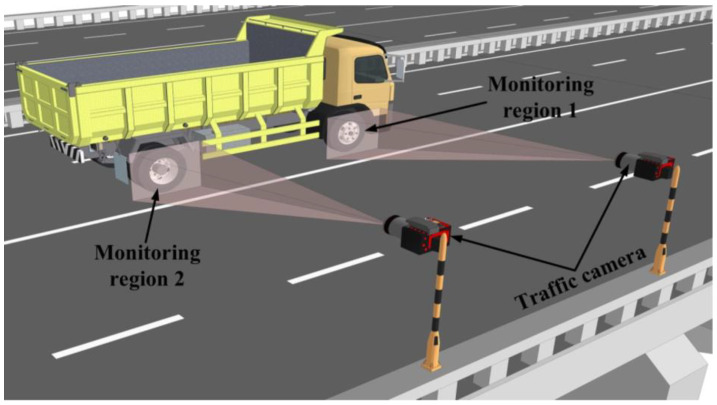
The vehicle weight identification system based on CV techniques [32].

Some scholars took the vehicle tire as the target of load identification and attempted to identify the vehicle weight parameters. Feng et al. [255] introduced an innovative CV-based vehicle weigh-in-motion (WIM) method. The method is based on simple physics: the tire–roadway contact force is equal to the contact pressure multiplied by the contact area. The area can be estimated by measuring tire deformation parameters such as tire–roadway contact length and tire vertical deflection using CV techniques, while tire pressure can be obtained from onboard sensors. Feng et al. [256] applied edge detection and optical character recognition (OCR) technology to identify the marking texts on the tire sidewall such that the manufacturer-recommended tire inflation pressure can be found, thus obtaining the vehicle tire brand, tire model, and tire size. This indicates that CV techniques such as edge detection and OCR are applied to enhance the measurement and recognition accuracy. Kong et al. [32] proposed a non-contact vehicle weight identification method based on the tire–road contact model and CV techniques, as presented in Figure 13. The theoretical model of tire–road contact was established based on the improved Hertz contact theory. CV techniques, including image segmentation and character recognition, were used to identify tire deformation and inflation pressure. Subsequently, Kong et al. [31] analyzed the tire–road contact mechanism, and numerical analyses were conducted to develop the tire contact force equations. The methodology for identifying the tire–road contact force by combining the derived equations and CV techniques was verified with field experiments on passenger cars and trucks. The experiment showed that the results predicted using the proposed method were in good agreement with the measured results. Compared with the traditional method, the developed method based on tire mechanics and CV has the advantages of high accuracy and efficiency, easy operation, low cost, and does not require the placement of sensors; thus, it provides a new approach to vehicle weighing.

### 4.4. Vehicle Multi-Parameters

In practical applications, it is necessary to obtain multiple parameters of the vehicle simultaneously, not just the above-mentioned single parameter, to meet the needs of systems such as intelligent transportation and SHM. For example, the type, length, number of axles, speed, trajectory, spacing, axle weight, and the total weight of the vehicle on the bridge are of great significance for bridge load statistics, condition monitoring and safety assessment.

Considering vehicle multi-parameter identification, Zaurin et al. [257,258,259,260] proposed a new method of SHM for bridge systems based on CV. An experimental study showed that the vehicle models could be effectively detected, classified, and tracked from the surveillance video. Zhang et al. [212] used the CV technique and Faster R-CNN algorithm to realize the identification of vehicle type, vehicle length, number of axles, vehicle speed and lane. Jian et al. [261] proposed a traffic sensing methodology that combines a DL-based CV technique with the influence line theory. The obtained results showed that the proposed method could automatically identify the vehicle load and speed with promising efficiency. Pan et al. [262] adopted the histogram of oriented gradients (HOG) and a random forest (RF) classifier for fast vehicle classification, and the vehicle speed and the vehicle–barrier separation distance were determined. Xia et al. [263] combined the network of strain sensors and CV to monitor the traffic load in short and medium-span bridges. The field monitoring results showed that this method can be used to identify the key parameters such as weight, speed, number, type, and trajectory of vehicles in a complex traffic environment. Dan et al. [264] proposed an improved full-bridge traffic load monitoring (TLM) method based on the YOLO-v3 convolutional neural network. The results showed that the vehicle trajectory, body, and tail contour of the vehicle can be more accurately identified using this method. Appathurai et al. [265] developed a novel hybridization of an artificial neural network (ANN) and oppositional gravitational search optimization algorithm (ANN-OGSA)-based moving vehicle detection (MVD) system. The system can be used for vehicle tracking, counting, vehicle speed measurement, and vehicle classification. The vehicle multi-parameters identification system based on CV and DL is an important bridge SHM and intelligent transportation system and a direction for future development.

## 5. Conclusions

This paper reviews the recent developments in CV-based bridge inspection and monitoring technology, including surface defect detection, vibration measurement, and vehicle parameter identification. The main conclusions and future challenges are as follows.

(1) Due to the lack of open-source datasets of bridge surface defects, researchers need to perform time-consuming and labor-intensive work for data preparation. The establishment of public datasets with generalization capability is urgently needed to solve the automated bridge surface defect detection [41].
(2) CV-based surface defect detection system is susceptible to its own hardware and external environmental factors. That is, hardware and environmental factors can adversely affect the performance of the detection system [65].
(3) It is difficult for existing DL algorithms to realize real-time detection of bridge surface defects. The computational efficiency needs to be improved, although some improvement has been realized through lightweight DL models [102].
(4) Most of the current research focuses on how to utilize advanced algorithms to identify surface defects on the bridge, while the assessment of their severity requires further research.
(5) Different robot platforms for surface defect detection have been developed for different bridge components. The development of a robot platform for surface defect detection with many applicable scenarios and low cost should be the focus of future research.
(6) Existing target-tracking algorithms are only suitable for scenarios with a large amplitude of vibration displacement, and struggle to obtain small vibration measurements of bridge structures. However, the amplitude of vibration displacement of rigid structures such as small and medium-span bridges, short cables, and short suspenders under environmental excitation is very small, and it is difficult for general target-tracking algorithms to accurately obtain the displacement time-history of bridge structures based on small vibrations.
(7) The existing bridge displacement monitoring method based on the CV technique has the problems of limited camera sampling rate and insufficient resolution in engineering applications. The development of a bridge displacement monitoring method based on the fusion of vision displacement and acceleration requires further research.
(8) Most of the methods in the existing literature can only obtain the mode shape profile of the bridge and cannot perform quantitative analysis of the mode shape. In addition, the existing research mainly focuses on the identification of discrete mode shapes of bridges, and the identification of full-field mode shapes is in need of further exploration.
(9) The vehicle parameter identification based on CV is mainly focused on the single-parameter identification of the vehicle, and there are relatively few studies on multi-parameter identification. With the continuous progress of CV technology, comprehensive multi-parameter identification of vehicles is a trend that will continue to grow in the future.
(10) The current bridge SHM systems mostly perform independent measurements of vehicles and bridges, and most of the assessment of bridge conditions is achieved only through the analysis of the output responses of the bridge, lacking accurate input information. Simultaneously obtaining the parameter information of the bridge structure and the vehicles on the bridge is an important direction for the development of the bridge SHM system in the future.

## Figures and Tables

**Table 1:** Open-source datasets of surface defects in bridges.

Name	Datasets Description	Application Fields
Aft_Original_Crack_DataSet_Second [71]	2068 bridge crack image with 1024 × 1024 pixels, all containing cracks	Semantic segmentation and object detection
Concrete Crack Images for Classification [72]	40,000 concrete surface crack images with 227 × 227 pixels, some without cracks	Semantic segmentation and image classification
SDNET2018 [73]	56,092 images with 256 × 256 pixels of bridge cracks, defects, and holes, some without cracks	Image classification
Crack dataset [71]	6069 images with 224 × 224 pixels of bridge cracks, all containing cracks	Semantic segmentation and image classification
Deep Crack [74]	8592 images with 256 × 256 pixels of concrete surface cracks	Semantic segmentation

**Table 2:** Summary of robot platforms with different functions.

Type of Robots	Author	Locomotion	Sensors	Defect Types
Mobile robots	La et al. [103]	Ground-based movement	Camera	Crack inspection
	Xie et al. [104]	Ground-based movement	Camera	Crack inspection
Wall-climbing robots	Sutter et al. [111]	Vertical climbing	Cameras	Concrete crack inspection
	Liu et al. [112]	Vertical climbing	Camera	Concrete crack inspection of bridge piers and towers
	Peel et al. [113]	Vertical wall climbing	Camera	Bearing inspection of bridges
	Nguyen and La [114]	Inclined wall climbing	Camera, hall-effect sensors IR IMU, Eddy current	Fatigue crack inspection of steel structures
Cable-climbing robots	Xu et al. [115]	Inclined cable climbing	Cameras, MFL sensors	Inspection of cable surface and inner defects
	Yun et al. [116]	Inclined cable climbing	Three cameras	Inspection of cable surface defects
	Cho et al. [117]	Vertical cable climbing	Camera	Surface defects inspection of vertical hangers
Aerial robots	Kang and Cha, [118]	Aerial flying	Camera	Surface crack detection of concrete structures
	Seo et al. [119]	Aerial flying	Camera	Concrete spalling and steel corrosion inspection
	Ellenberg et al. [120]	Aerial flying	Infrared cameras	Subsurface delamination inspection
	Sanchez-Cuevas et al. [121]	Aerial flying and physical contact	Camera, supersonic sensors	Crack depth measurement
Multipurpose drones	Jiang et al. [122]	Aerial flying and wall climbing	Cameras	Real-time crack length and width measurement

**Table 3:** Comparison of different target-tracking algorithms.

Method	Structure	Measurement Distance (m)	Parameters	Maximum Error	RMSE (mm)	Field Condition
***Template matching***						
OCM [21]	Cable	–	Frequency	2.81%	–	Good weather
Zero mean NCC [141]	Pedestrian Baker bridge	55.3	Frequency	8%	–	Overcast
Zero mean NCC [142]	Railway bridge	6.9	Displacement	–	0.35	Sunny
OCM [143]	Manhattan bridge	300	Displacement	–	–	Good weather
***Feature matching***						
SIFT [144]	Bridge	9.7	Frequency	2.5%	–	Partial shading
Hessian + SURF [145]	Suspension bridge	71.2	Displacement	–	0.03	Low-speed winds
***Optical flow***						
Virtual visual method [146]	Pedestrian bridge	–	Displacement	–	–	Good weather
Motion magnification [147]	Arched bridge	30	Frequency	6.79%	–	Sunny
Harris corner detector + KLT [148]	Pedestrian bridge	3–4	Frequency	1.51%	–	Low-speed wind
Motion magnification [149]	Bridge	80	Frequency	6.25%	–	Light drizzle
Harris corner detection + KLT [150]	Steel truss bridge	4.6	Displacement	–	2.14	Indoor

## Data Availability

Data sharing not applicable.
